# A qualitative case study of pregnancy and early parenting in Canada’s federal prisons for women

**DOI:** 10.1371/journal.pone.0294961

**Published:** 2023-12-27

**Authors:** Martha Paynter, Ruth Martin-Misener, Adelina Iftene, Gail Tomblin-Murphy

**Affiliations:** 1 Faculty of Nursing, University of New Brunswick, Fredericton, New Brunswick, Canada; 2 School of Nursing, Dalhousie University, Halifax, Nova Scotia, Canada; 3 Schulich School of Law, Dalhousie University Halifax, Nova Scotia, Canada; 4 Nova Scotia Health Authority Provincial Office, Nova Scotia Health Halifax, Nova Scotia, Canada; University of Greenwich, UNITED KINGDOM

## Abstract

**Objective:**

The aim of this study was to understand the experiences of pregnant people and new parents in Canadian federal prisons for women, and to better understand their ability to participate in the institutional Mother Child Program.

**Methods:**

This qualitative case study used semi-structured interviews with people who experienced federal incarceration during pregnancy or the early parenting years.

**Findings:**

Major themes in the analysis include: 1) Reasons why- and why not- to participate in the Mother Child Program; 2) Mothering from inside; 3) Health care; and 4) Strategies and survival.

**Key conclusions:**

Mothers describe multiple reasons for choosing not to participate or being ineligible for the Mother Child Program; separation as common and traumatic; health services as inadequate; and mental health concerns being met with punishment. Alternatives to incarceration are recommended.

## Introduction

The number of people admitted to Canadian federal prisons designated for women increased 16.3% between 2009 and 2019 [1]. Black and Indigenous people are disproportionately incarcerated: 8% of prisoners are Black and 32% are Indigenous [2]. Among federally sentenced women, approximately 50% are Indigenous [3]. Increasing incarceration of women and people who can become pregnant has serious consequences for reproductive health and parenting. In the early 2000s, Correctional Services Canada (CSC) launched the institutional Mother Child Program (MCP) to mitigate the harm of separating mothers from their children [4]. The program has never been evaluated [2], and health outcomes associated with the program have never been investigated [5]. The objective of this study was to explore the experiences of federally sentenced pregnant people and parents of young children and whether or not they participated in the MCP. Note: this paper uses gender inclusive language, acknowledging that people who have been pregnant or parenting while federally incarcerated may have diverse gender experiences and identities. Where citing a source that uses the gendered terms, those are used. As all participants identified as mothers, the term mother is used when discussing findings.

Commissioner’s Directive (CD)-800 governs health services in the federal prison system. CSC health professionals will “provide health services to offenders consistent with relevant provincial/territorial and federal legislation, the provincial/territorial regulatory body’s professional practice standards, as well as CSC policies and practice directives.” Pregnancy is only mentioned once, “For pregnant offenders, Health Services will ensure arrangements for childbirth are made at an outside hospital.”

Commissioner’s Directive 768 sets out the responsibilities, eligibility, requirements, and processes for the MCP across the six federal prisons for women. Children under five years of age can live full-time, on-site, with their parent in a minimum-security living unit with adjoining rooms; children under seven years of age can live on site part-time. Children may leave the institution for custody arrangements, visits with other family, and health and recreational services. Prisoners must not have been convicted of an offense against a child and must be classified as minimum or medium security risk. Participation depends on completing courses such as infant first aid and CPR, car seat safety, or parenting. Peer “babysitters” must be available, as well as a trusted adult in the community nearby to retrieve the child in an emergency. Other prisoners who may access the mother-child living unit are screened. Provincial child protection services must assess the appropriateness of the parent and make a recommendation to the warden.

Incarceration impinges reproductive autonomy through routine use of strip searching, isolation in solitary confinement, physical restraints, and exposure to increased risk of sexual assault [6–10]. Research has found CSC fails to attend to differences of gender, race and other health factors when assessing security classification, resulting in inflation of women’s risk profiles and escalated restrictions on liberty [11]. Health needs such as PTSD are interpreted as security risks, and psychological illness is perceived as “madness” in need of coercive regulation, segregation or medicalization [12–14]. Mothers separated from their children due to incarceration experience severe loss [15, 16]. Jailed parents are three to five times more likely to experience depression compared to the norm, with higher rates among mothers [17, 18].

The children of incarcerated parents in Canada are described as an “invisible” population [19]: uncounted and unaddressed by policy [20]. Who cares for children while their parents are incarcerated in Canada is not known. A recent U.S. study found most children of incarcerated mothers live with their grandparents [21]. In Canada, the disproportionate incarceration of Indigenous women coincides with disproportionate numbers of Indigenous children in foster care [22].

For over 70 years, researchers have recognized keeping primary caregivers and children together enhances childhood attachment and emotional health [23]. Proponents of MCPs believe keeping the mother-child dyad together prevents psychological, physiological, and developmental harm to the child [24]. International reviews of health research related to Mother Child units and prison nurseries have not included any studies based in Canada [5, 25]. A 2014 study by Brennan found the MCP to be infrequently used due to 1) strict eligibility criteria; 2) prison overcrowding; 3) limitations of the physical prison environment; and 4) the increasingly punitive nature of corrections [26]. Miller (2017) finds Indigenous women more likely to receive higher security classification making them ineligible for the MCP [27]. From 2001–2018, the MCP had only 133 participants, an average of seven per year [28]. There was no correlation between the size of each prison and the number of people in the MCP. Non-Indigenous people were more likely to participate than Indigenous people.

To justify continuation, changes, or expansion of the MCP in Canada, evidence is needed regarding the impact of eligibility restrictions, rates of participation, and to what extent the MCP promotes parent and child health. The purpose of this study was to begin to address these gaps. The research question guiding this study asks, what are pregnant people and new parents’ experiences of federal incarceration, whether they were able to participate in the MCP or not?

## Materials and methods

### Theoretical background

Abolition feminism informs the theoretical foundation for this study. Incarceration impinges on reproductive rights while perpetuating cycles of racism, colonialism, and capitalism and amplifying violence and harm [29, 30]. Federal prison is an enormous public expense in Canada- over 1.63 billion dollars/year [31]. Abolitionists advise redirection of investment to create systems of support, including housing, health services, food security and other types of care that prevent experiences of criminalization and avoid the need for health services within the punitive operations and infrastructure of prison [32]. Abolition critiques MCPs for normalizing incarceration, increasing the need for public spending on incarceration, and crowding out alternatives such as housing.

### Researcher’s position

The first author has volunteered in prisons for over ten years and facilitated reproductive health and justice workshops for the Canadian Association of Elizabeth Fry Societies (CAEFS) in the five English language prisons for women in Canada [32].

### Design

This study uses a qualitative case study design, a comprehensive strategy encompassing formulation of the research question, data collection, analysis, and presentation of findings [33]. Case study can be used to understand complex social phenomena addressing “‘how’ and ‘what’ questions when the researcher has little or no control over the phenomenon of interest” [33]. This case study began with evidence-informed assumptions to guide the data collection, focus the analysis, and shape the discussion [33, 34]. These included:

1. The MCP is infrequently used [26, 27].
2. MCP participation is a positive experience [35].
3. For incarcerated parents, separation from children is traumatic [15].
4. Perinatal health care provided by CSC is inadequate [1].
5. Parents use agency and solidarity to navigate the experience.

### Setting

The setting is the Canadian federal prison system designated for women. The six prisons are in Abbottsford, BC; Edmonton, Alberta; Maple Creek, Saskatchewan; Kitchener-Waterloo, Ontario; Joliette, Quebec; and Truro, Nova Scotia. To mitigate foreseeable challenges with recruitment, the first author collaborated with CAEFS member societies to recruit participants.

### Study participants

This study used criterion-based sampling and aimed for diverse experience and representative among participants with lived experience of federal incarceration while pregnant or parenting young children [36]. Interviews were conducted in either English or French in January—May 2020. The sample size was not predetermined and continued until data saturation was achieved [37]. Study participants varied in age, cultural background, life experience and geographic location. All identified as cisgender women.

### Data sources and collection

The first author conducted semi-structured interviews in person or over the phone which were audio recorded and professionally transcribed verbatim. The two French interviews were professionally translated into English. The interviews incorporated both open-ended and focused questions about a) participation- or not- in the MCP; b) being pregnant or having a young child while federally incarcerated; c) health care; and d) what was helpful and what they would recommend.

### Data analysis

Thematic data analysis [38] was used to develop themes. The first author read all transcripts several times and created an organized archive. They were reflexive about how experiences and relationships prior to and after the data collection influenced interpretation, keeping a written journal and frequently debriefing with the research team. Using Microsoft Word and Excel applications for data management, the first author coded the transcripts to address the main concepts. They compared codes with the research questions and the assumptions to develop a coding matrix. A peer independently coded 10% of the transcripts using the matrix. The two coders developed themes deductively and inductively, determining both data alignment with existing concepts and refining themes using respondents’ language.

### Trustworthiness

The first author used processes developed by well-established qualitative researchers to support trustworthiness [39]. The research question was clearly articulated, assumptions were constructed, the design and sampling strategy were appropriate, data was collected and analyzed systematically. Credibility was established through prolonged engagement and researcher triangulation. Thick descriptions of the findings, offering both description and interpretation, delineate limitations to transferability. There are findings relevant to the federal prison context that do not apply to other custodial contexts. Dependability is supported through extensive documentation. The first author maintained a continuous audit trail, an orderly, anonymized log of where, how and with whom each interview was conducted, as well as decisions and steps taken to advance the research.

### Ethical considerations

This study was approved by the Dalhousie University Research Ethics Board #2019–4937. All participants were provided with a consent form in advance of the interview, and people who were interviewed by phone provided verbal consent. Verbal consent was obtained by the researcher reviewing the consent form verbally with the participant and then reviewing a verbal consent script with the participant. Once obtained, verbal consent was recorded by the researcher with a date and signature. This process was approved by the Research Ethics Board. To minimize coercion, it was made explicit that participants can stop or leave the interview at any time, and that the honorarium would be distributed regardless of if the interview was completed.

## Results

### Participant demographics

Participants ranged in age from early 20’s to into the 60’s. One self-identified as Black, one as Indigenous, two as Francophone and one as 2SLGBTQIA+. Four had first been incarcerated at Nova Institution, and there was one participant from each of the other five federal prisons. One started their sentence in the 1990’s, one in the 2000’s, four in the 2010’s and three in the 2020’s.

### Themes

For most mothers, participating in the MCP was out of reach, and they planned alternative child care. They felt profound, often debilitating trauma in separation. They experienced restricted access to health services, neglect of their emotional distress, and punitive treatment. Self-advocacy and peer support helped them cope.

### Theme 1: Reasons why- or why not- to join MCP

Mothers were concerned about eligibility and whether to apply to the MCP. Of the two MCP participants in the sample, both had been pregnant while federally incarcerated, and both disclosed they had served long sentences. Neither identified as Indigenous or a person of colour or 2SLGTBQIA+, and one was francophone. Participant described how MCP eligibility was determined by your institutional record, such as security level, crime, and child protection history. While consistent with the eligibility restrictions outlined in CD-768, in practice, participation appeared to be more complicated. For instance, both MCP participants had supportive spouses living in the same town as the prison. Both felt their long sentences gave them time to get to know how to navigate the system to get their needs met.

Several mothers knew they were ineligible for the MCP due to security classification. Several said they wanted to participate, would have been eligible, but did not have support in the application process. The mothers who were not pregnant while in the prison were never offered the MCP opportunity, although the MCP is open to any children under age five. Some had already had their children removed by child protection services. Once COVID-19 hit, some believed the MCP was closed- although it had not. For these reasons, only a “lucky few” participated.

Some mothers resisted bringing children into the prison environment which they felt was unsafe and unpredictable. One described the conflict she felt:

I was torn because I would have really liked to have [child’s name] with me but then I thought about it, and I thought with certain people in there and stuff I’d probably rather not have my child in that environment because it’s so volatile and there’s certain people did certain crimes that I wouldn’t want my children around. I just, you know I think it’s a great program but it’s also not a great program. [P9]

In 2019, Samantha Wallace, a 28-year-old mother of four, died while incarcerated at Nova Institution [40]. Wallace’s youngest child was just four months old at the time, living with Wallace’s sister. Several participants mentioned the story of Samantha Wallace. For one mother, Wallace’s death was proof the prison was an inappropriate place for a child.

[Wallace] was complaining how sick she was, why would anybody want a child in jail? They don’t have a clean space, they don’t have, like, they have people that are around you that shouldn’t be around children for one… So, you’re kind of forced to not have your child there. [P12]

Some mothers chose not to participate in the MCP because they did not want to be under additional surveillance, not only from prison staff, but also from provincial child protection services. Prior to incarceration, many had already experienced child protection involvement- including in their own youth. Others had avoided child protection up to that point and wanted to protect their families from it. Mothers worried that even if they qualified for MCP, participation was tenuous. If something went wrong in the prison, your child could be taken from you. As one respondent recounted, “There was one woman…she was actually segregated for something that she didn’t even start and her child was taken out of the program.” [P9]

Some mothers were relieved to have had the power to arrange for the care of their children in community. Knowing children were safe and cared for and having autonomy in organizing that brought them peace. One mother permanently placed her child in the care of a cousin who had tried for years to have a child. To her, this was “giving back”, in keeping with her Indigenous culture.

I had every intention of bringing him into the Mother Child Program once I got to [prison] so I had my minimum security [classification], everything was good. But then I started like being really in-touch with my culture [Indigenous] and learning my traditions, doing a lot of sweats, a lot of, I moved to the healing house, did a lot of one-on-ones with [Elder name] and I came to the decision not to bring him in with me because I felt in our culture it’s a lot to do with energies, a lot to do with giving back. [P12]

### Theme 2: Mothering from inside

The second major theme addressed traumatic experiences as mothers in prison. Separation caused isolation, loneliness, and emotional distress: “It was definitely a hard adjustment… it was definitely a terror, was definitely lonely, it was scary, it was depressing, traumatic, it was probably the scariest time of my life.” [P22] For some, the existence of the MCP made the situation worse: seeing other mothers with babies prompted feelings of jealousy and pain. For others, the separation was layered on top of earlier traumas including childhood abuse and domestic violence. Harsh words from prison staff exacerbated the trauma: “They kept telling me that my daughter would never be around me and that I would not have help on the outside, that I lost my daughter for good and that I will never see her again and everything.” [P20] Some recounted experiences of sexual violence from officers in police custody, provincial jail, and federal prison. One described being brought to the police station while she was breastfeeding, and having her bra forced off. Another was sexually assaulted by a correctional officer. Several were strip-searched, which one described as, “so dehumanizing, you feel like just an animal, right?” [P12]

For those in the MCP, their mothering and children were under surveillance. One MCP participant described how babies’ weights were monitored weekly for the first year, and failure to gain weight could result in child removal:

At [prison] when the baby loses a bit of weight, they take him away from his mother. Even if it’s just a few ounces they [Child Protection] take him away from his mother… They put them with families. And I saw a lot of mothers whose children were taken away because their weight went down a bit…They just took the child away and sent them away to a foster family [P18]

Peers would report to staff about baby’s noises, or staff themselves would complain:

Well, when the officers went by for the count they’d say, “Are you going to keep your kid quiet?” Yeah well, listen, if my child is crying it’s for a reason, he’s hungry, there’s something. I’ll feed him soon, after the count.” “But are you going to shut him up?” Because he’s crying. I’m not about to shut him up. I’m not going to put my hand over his mouth to shut him up. [P18]

One had a peer try to “sabotage” her, by writing complaints to the parole board and child protection services. She was resolved not to lash out in anger for fear of punishment or being labelled as difficult: “When you’re the mom in the Mother Child Program, even if you’re not the one in the wrong, if you react, you’ll be held responsible regardless. [P11] Because of the risk of punishment or being reported to child protection services, mothers described hiding their needs for support from prison staff.

Although contact with children, especially by phone, was a lifeline for the mothers, it could also cause its own traumas. Calls were expensive, and loading money onto a phone card was only permitted once monthly. For everyone, calls were subject to surveillance by prison staff. If staff overheard something they disapproved of, they could–and sometimes did- report it to child protection services. Mothers would refrain from expressing how they really felt. And some mothers chose not to call anymore, to avoid the emotional toll.

The mothers rarely received visits, not only because of physical distances from children, but because of the lengthy, complex approval process- and reliance on people on the outside. For example, one mother required her children’s two dads to both approve before their grandmother could bring them in. COVID-19 put a halt on in-person visitation, and even video visits were difficult as children had to go to a CSC-approved centre. One respondent complained about not getting to have a birthday video visit due to staffing insufficiency, and as a result, had her video visits suspended for a month.

Another mother said child protection services discouraged visits from children in foster care, to facilitate a “clean break” and placement in permanent care. She said, “There’s no mother who’s ever having a clean break from the kids and her oldest kid is ten years old, you’re not clean breaking him from his mother, or any of them for that matter. There’s no clean break to take a mother from her children. [P7]

### Theme 3: Health care

Experiences of healthcare varied across participants. The most recent MCP participant described CSC perinatal health services as positive and adequate, and felt that they were well supported in their pregnancy:

They took care of that by really checking with me, seeing a psychologist, behavioural counsellors… they were really good in that aspect, monitoring my wellbeing cause they know anxiety is really hard in the beginning of three months of pregnancy. [P11]

She was also provided with doula support. The doula provided postpartum support, helped her feel more secure in parentings, and encouraged and facilitated breastfeeding initiation:

I didn’t want to breastfeed, and the doula changed my mind. I was totally supported in doing it. [P11]

She felt the prison staff anticipated and addressed her needs, but pregnant prisoners who were not accepted into the MCP did not get the same level of support. By contrast, the other MCP participant who had been in the program years before at another institution, said she received “no information” about pregnancy from the health care staff, and had to purchase her own prenatal health book. She used maxi pads when denied diapers. Several months into breastfeeding, she developed mastitis, and said the health staff did not know how to help her, so she had to stop feeding her child.

Outside of the MCP, the mothers reported inadequate support for reproductive mental health, and operational barriers to access CSC health services. For example, waiting in long lines for medication- even outdoors- while exhausted from pregnancy. Many reported staff not showing up to appointments, and not providing notification:

I make an appointment, I show up, the health care or counselling is not there. They never called me to you know follow-up to be like oh sorry I can’t make it in today, it’s just like you just kind of figure it out for yourself, o.k., they’re not in I’ll wait for a slip or a piece of paper in my mailbox for another date. It’s just really slack. [P22]

Mothers relied on staff escorts for external appointments, and storm days, staff illness, or short staffing could interfere. While accompanied by correctional officers, respondents faced privacy violations. One described the humiliation of having correctional officers present for a vaginal exam. Another was handcuffed and “paraded” through the hospital.

The wait-times for speciality services could be years-long. Many felt the prisons over-relied on pharmaceutical approaches, and declining medications was interpreted as disobedience. Yet health care staff also disapproved when they sought medication, calling them “drug seeking” [P9]. Several felt they were not believed by nurses, and disrespected:

[The CSC nurses] tell your business in front of other people, they have no confidentiality… They’ll tell you more or less who has HIV. Like “stay away from them,” if you know what I’m trying to say, they won’t say it straight out but they’ll say it to you in a way [P12].

Participants described how mothers may engage in self-harm behaviours as a reaction to the prison environment. Prison staff interpreted distress not as a health issue needing care but as a negative “behaviour” warranting punishment:

They become self-harmers inside while they’re incarcerated because they don’t have any other avenue. And what’s their solution to that? Put them upstairs and lock them up… they’ll put them in a room on camera status, so they strip them of their clothes, they give them a little gown…and they put them in there, no mattress, no nothing. [P9]

### Theme 4: Self-advocacy and survival

The mothers survived through strategic, persistent self advocacy and peer support. Although camaraderie with other women made a difference, solidarity and sharing were discouraged by the institution. Long periods of institutionalization led the two MCP participants to feel they had rapport with staff and could advocate for in non-adversarial ways. But some mothers had filed grievances, complaints, human rights cases and even civil lawsuits.

Some described the official pathways for complaint as ineffective, “I’ve put countless grievances in. I’m the grievance queen. I could build a house out of all the grievances I’ve put in…They deny them. They say oh no it’s not a founded grievance.” [P9]

In some ways, being a mother was motivation to speak up, but also more reason to be careful about it to avoid punishment like loss of video visits. One described how, “You have to take something from yourself every day to swallow your pride over and over again with people putting you down and you can’t say anything because then you’re being disrespectful, you’re being defiant.” [P12] Another said she kept her head down and avoided confrontation, “I kept to myself a lot, I minded my ps and qs, I did everything I was supposed to do, I didn’t start any trouble, so everything was, everything was pretty much smooth sailing for me, and I got the respect that I gave.” [P22]

When asked about what would make things better, the mothers had a few recommendations to improve experiences, such as making the MCP more accessible- and available until children were older. The participant who had doula support recommended doulas for everyone. Several advocated for changes to visiting and phone policies to protect children from cancellations and remove financial barriers. Many recommended expanding mental health supports, such as peer counsellors, and access to independent health professionals. They recommended the best way to deal with being a mom in prison was to not be one: “I counsel women who are pregnant to stay out of prison. Because, no, it’s not a liveable situation when you’re pregnant, being in a correctional facility.” [P18]. Instead, many expressed the answer lay outside of prison:

Some people definitely can change without being sent to prison and having to deal with separation from their kids…if I could change the fact of women being incarcerated, like moms being incarcerated I would definitely choose for them not to go to jail. [P22]

## Discussion

In alignment with previous scholarly work, this study identifies serious problems with the MCP include challenging and often opaque eligibility criteria, surveillance, and uncertain outcomes [41, 42]. The MCP is inequitably available, qualifying is uncertain; and continuous participation is precarious. Neither of the two MCP participants identified as Indigenous; this aligns with findings that Indigenous women face increased barriers to participation and higher security classification [11, 27]. Perinatal health care provided while incarcerated is inadequate. CSC health professionals failed to adhere to professional standards of practice to protect confidentiality, dignity, and to exercise compassion [43].

This study identifies several ways that parental incarceration drives reproductive oppression and is deleterious to child and adult health. The MCP is an inadequate solution to parental incarceration, the trauma of separation, and the denial of care. The steadily increasing numbers of people in prisons designated for women, high lifetime parity, and high rates of unplanned pregnancy point to the urgency and importance of addressing perinatal health and planning for the care of young children when parents face federal sentences. This study may help to inform health professionals caring for incarcerated people and to identify priority areas for policy development and future research addressing parental and maternal incarceration. Stemming from our findings, we make several key recommendations:

1. ***The Mother Child Program*:** A formal, external, independent evaluation of the federal Mother Child Program is necessary. CSC must collect data on the number of children affected by federal incarceration.
2. ***Sentencing*:** Suspension of custody should be considered to allow mothers to care for their children in community and avoid prison. The negative impacts on children of parental incarceration health should be considered in sentencing decisions.
3. ***Health Care*:** Health professional educators must integrate prison health into training to prepare clinicians for the challenges of caring for incarcerated people. Health professionals in prisons must resist threats to ethical care. Health professionals in community must be aware of prison procedures including strip searches, restraints, and discipline.
4. ***Agency*:** The impact of incarceration on both adult and child health must guide the development of meaningful alternatives to prison that recognize parental strengths and provide useful support. As was adeptly said, “People just need help, and they need that support to get the help so you know prison definitely isn’t always the answer” [P22].

### Limitations

There are several limitations to this study. All interviews were conducted in English or French, restricting who could participate. Many interviews were conducted by phone and nuance, body language and depth may have been lost. Although Elizabeth Fry Societies greatly supported recruitment, the sample size is small. Only two MCP participants limits conclusions about the MCP. Although Black and Indigenous people are disproportionately incarcerated, few study participants disclosed Black or Indigenous identity. Racism and colonialism likely affect experiences more broadly than our findings delineate.

### Strengths

Despite limited sample size, study participants exhibit many commonalities and differences, and studying across differences enhances robustness of findings [33]. The themes incorporate contrast and provide a start towards theoretical replication and external validity. This study prioritizes the voices of people with lived experience.

## Conclusions

This study found participation- or not- in the MCP played a critical role in determining the experiences of federally incarcerated pregnant people and people with young children. Barriers to the MCP include lack of information about the program, exclusionary eligibility criteria, and fear of exposing children to an unsafe environment. Because access is so unequal, the MCP is a flawed solution to escalating numbers of incarcerated parents. Incarceration exposes parents to traumas of separation and institutional dangers including sexual violence. Parental efforts to stay connected to their children were largely limited to telephone contact. Health care involved routine violations of privacy, confidentiality, excessive waits, inadequate access to mental health services, and an over-reliance on pharmaceuticals. Postpartum distress was not recognized as a health care need and was met with punishment. Despite the strictly regulated experience of incarceration, respondents used their agency, creativity, strategic thinking, and solidarity to navigate the prison and support themselves and each other.
